# Risk Factors Affecting Muscle Mass Decline in Maintenance Hemodialysis Patients

**DOI:** 10.1155/2022/2925216

**Published:** 2022-12-20

**Authors:** Yiqi Song, Qian Zhang, Li Ni, Minmin Zhang, Mengjing Wang, Weichen Zhang, Jing Chen

**Affiliations:** Division of Nephrology, National Clinical Research Center for Aging and Medicine, Huashan Hospital, Shanghai Medical College, Fudan University, Shanghai 200040, China

## Abstract

**Objective:**

There is a high prevalence of sarcopenia in maintenance hemodialysis (MHD) patients, which is known to be associated with increased mortality. This study is aimed at analyzing the risk factors affecting muscle mass decline in MHD patients.

**Methods:**

This retrospective study included MHD patients who underwent two body composition assessments in October 2013 and November 2017. Depending on whether there was muscle loss or not, the patients were divided into a normal muscle mass (NMM) group and a muscle mass decline (MMD) group. According to the muscle mass decline rate, patients in the MMD group were further classified into a low-rate group and a high-rate group. Biochemical variables, serum vitamin concentrations, anthropometric data, SGA, muscle mass, handgrip, and daily steps were assessed. Risk factors for muscle mass decline were screened by multivariate logistic analysis and linear regression analysis.

**Results:**

Of the 72 MHD patients included in this study, 33 were male and 39 were female with a mean age of 56.80 ± 10.86 years and a mean dialysis duration of 7.50 ± 5.20 years. Age (*P* = .014) and serum 25(OH)D (*P* = .040) were found to be associated with a higher risk of muscle mass decline after adjusting for gender, dialysis vintage, albumin, and hs-CRP (*P* = .040). Further analysis found that dialysis vintage (*β* = 0.285, *P* = .030), 25(OH)D (*β* = −0.351, *P* = .007), and log NT-proBNP (*β* = 0.312, *P* = .020) were risk factors associated with the muscle mass decline rate in MHD patients.

**Conclusion:**

Age and serum 25(OH)D were associated with a higher risk of muscle mass decline, while 25(OH)D, dialysis vintage, and NT-proBNP were associated with the muscle mass decline rate in MHD patients.

## 1. Introduction

Protein energy wasting (PEW) is a common complication in patients with end-stage kidney disease (ESKD), which not only affects the quality of life but also associates with high mortality [1]. Despite combination therapy with optimization of dietary nutrient intake and dialytic regimens plus appropriate treatment of metabolic disturbances such as metabolic acidosis, systemic inflammation, and hormonal deficiencies, ESKD patients still potentially have an increased risk of PEW (18-75%) [2].

Muscle wasting is the major feature of PEW [3]. Preservation of muscle mass is an essential goal in the management of PEW in maintenance hemodialysis (MHD) patients [3]. Several mechanisms that improve muscle metabolism have been identified, such as activation of the ubiquitin–proteasome system (UPS), caspase-3, lysosomes, and myostatin [3]. However, there is still a lack of effective therapies for PEW. Many MHD patients suffered from continuous muscle mass decline. The reason for the decrease in muscle mass is worth further exploration.

Vitamin deficiency is common in MHD patients [4, 5]. An observational study showed that up to 97% MHD patients with ESKD had vitamin D insufficiency [6]. Beyond the well-known functions in bone metabolism and calcium–phosphate homeostasis, vitamin D also plays an increasingly important role in muscle metabolism [7]. In older persons, serum 25(OH)D levels are reduced in parallel to the severity of muscle functions [8]. Studies suggest that vitamin B12 deficiency may be related to sarcopenia in older adults [9]. Other reports found that 10-25% of dialysis patients have low plasma vitamin C levels (<10 mM), which is known to be associated with increased mortality [5]. Whether vitamins play a role in muscle mass decline in MHD patients remains uncertain. We therefore conducted a retrospective study to found out the risk factors which may affect muscle mass decline in MHD patients.

## 2. Methods

This retrospective study was performed in the hemodialysis center of Fudan University Huashan Hospital (Shanghai, China). The selection criteria were (1) ≥18 years; (2) thrice weekly hemodialysis for more than 3 months; (3) loss of residual renal function defined as a urine volume < 200 mL/24 hours; and (4) had two body index analysis (BIA) records between October 2013 and November 2017. Patients with severe inflammatory diseases, malignancy, cardiac and cerebrovascular events, and limb malformation were excluded from the study. Patients used LOPS15 (B. Braun, Melsungen, Hessen, Germany) for HD and HIPS15 (B. Braun, Melsungen, Hessen, Germany) for HDF. Patients were treated with HDF once every two weeks.

According to the two BIA records, patients who suffered reduced muscle mass were named muscle mass decline (MMD) group and others as normal muscle mass (NMM) group. Then, the MMD group was further divided into a low-rate group and high-rate group based on the median of muscle mass decline rate. All participating patients signed an informed consent and passed the ethical review by the Ethics Committee of the said hospital.

### 2.1. Biochemical Variables

Pre- and postdialysis blood samples were collected on a midweek day. All laboratory measurements were performed using standardized and automated methods. Normalized protein nitrogen appearance (nPNA) was calculated by using urea kinetic modeling (UKM) formulas [10]. Serum levels of calcium, phosphorus, intact parathyroid hormone (iPTH), N-terminal pro-B-type natriuretic peptide (NT-proBNP), albumin, hemoglobin, and cholesterol were collected. Serum vitamin levels were assessed using enzyme-linked immunosorbent assay (ELISA) kits. C-reactive protein (CRP) and serum bicarbonate were also observed.

### 2.2. Nutrition Assessment

Nutrition assessment included anthropometric measures and modified quantitative subjective global assessment (MQSGA) scores. Anthropometric measures included hipline, waistline, midarm circumference (MAC), and triceps skin-fold thickness (TSF). MAC and STF were performed on the nonfistula side limb. The average of the measurements was recorded after 3 times by the same observer. All measurements were performed after a dialysis session. Then, the waist/hip ratio and midarm muscle circumference (MAMC) were calculated and used in this study [11]. The anthropometric measures and MQSGA scores were assessed by the dietician.

### 2.3. Measurement of Muscle Mass and Strength

Body composition assessment was performed by bioelectrical impedance analysis (BIA) within 30 min after a midweek hemodialysis session. BIA measurements were conducted using a bioimpedance analyzer (TANITA Corporation, Tokyo, Japan), and whole body bioelectrical analyses were carried out using standard protocol as previously reported [12]. According to the BIA result, height-adjusted appendicular skeletal muscle mass (ASM/H^2^) was calculated by the following equation: ASM/H^2^(kg/m^2^) = ASM/Height^2^. Patients were divided into 2 groups according to percent change in ASM/H^2^: NMM group (≥0%) and MMD group (<0%). The muscle decline rate was calculated according to the two BIA records and intervals described as the following formula: Muscle mass decline rate(kg/m^2^/year) = (pre‐ASM/H^2^ − post‐ASM/H^2^)/interval.

Handgrip strength was measured with a handgrip dynamometer in the nonfistula side of the MHD patients before a dialysis session. The mean of the three measurements was used in this study. Digital pocket pedometer (Omron) was used to record daily steps. Daily steps were the mean of dialysis days and nondialysis days.

### 2.4. Statistical Analysis

Results are expressed as mean values ± standard deviation (SD) or percentage and quartiles for nonnormal distributions. Comparisons of mean values were made by the independent sample *t*-test for continuous variables and Pearson's chi-square test or Fisher's exact test for categorical variables by SPSS 23.0 software. A *P* value < .05 was considered statistically significant. Univariate regressions were firstly used; only variables with *P* ≤ .05 were included in the multivariate logistic analysis; and linear regression analysis was conducted to assess the associations between muscle mass decline and variables. Variables with *P* ≤ .05 were included in the multivariate linear regression analysis.

## 3. Results

**Figure 1:**
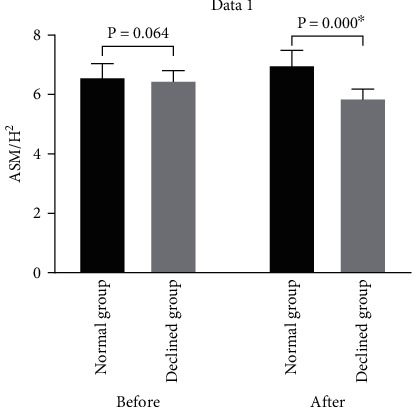
ASM/H^2^ change in the normal muscle mass group and the muscle mass declined group. Data are expressed as the mean ± standard deviation (SD). ^∗^*P* < .05, after 2.82 ± 1.23 years (post) versus at the start (pre).

A total of 72 MHD patients were included, of whom 33 were male and 39 were female, with a mean age of 56.80 ± 10.86 years and a mean dialysis duration of 7.50 ± 5.20 years. The main primary disease was glomerulonephritis (40.3%). Patients were followed up for a median 2.5 years (interquartile range: 1.6-3.8 years). The patients were first divided into a NMM group and a MMD group depending on whether they suffered muscle loss or not. Changes in ASM/H^2^ of the two groups are shown in Figure 1. The baseline characteristics were similar between the two groups (Table 1), and all laboratory parameters showed no significant difference except for age (*P* = .007) and 25(OH)D (*P* = .006) (Table 1). As for anthropometric indices, females showed a higher level of midarm muscle circumference than males in the NMM group (*P* = .012).

Risk factors associated with muscle mass decline were identified by logistic regression analysis (Table 2). The results showed that age (*P* = .014) and serum 25(OH) (*P* = .040) were associated with a higher risk of muscle decline after adjusting for gender, dialysis duration, albumin, and hs-CRP.

Patients in the MMD group were further divided into a low-rate group and a high-rate group according to the ASM/H^2^ decline rate (Table 3). The mean age of patients in the low-rate group was 55.77 ± 7.68 years vs. 62.40 ± 10.86 years in the high-rate group (*P* = .062). Patients in the high-rate group showed a longer dialysis duration (*P* = .016) and a higher level of log NT-proBNP (*P* = .006). In addition, the level of several vitamins was lower in the high-rate group, including serum 25(OH)D (*P* = .048), vitamin C (*P* = .017), vitamin B2 (*P* = .018), vitamin B12 (*P* = .048), and vitamin E (*P* = .047). There were no significant differences in albumin, hs-CRP, cholesterol, CO_2_-CP, nPNA, grip strength, and daily steps between the two groups. Multivariate linear regression analysis showed that dialysis duration (*P* = .030), 25(OH)D (*P* = .007), and log NT-proBNP (*P* = .020) were risk factors associated with the muscle mass decline rate in MHD patients (Table 4).

## 4. Discussion

Muscle mass has been found to be an important prognostic indicator of MHD patients [13, 14]. Hence, the dynamic changes in muscle mass are even more noteworthy [15, 16]. In this study, we found that age and serum 25(OH)D were associated with a higher risk of muscle decline, while 25(OH)D, dialysis duration, and log NT-proBNP were associated with the muscle mass decline rate in MHD patients.

Muscle mass is known to decline with age [17]. When a MHD patient reaches 65 years of age, muscle mass depreciates rapidly [18]. In this study, we found that age was associated with muscle mass decline in MHD patients, but not between low- and high-rate groups. This result suggests that in individuals who suffer severe muscle mass decline, there may be some other factors involved.

Albumin is known as a widely used marker for assessment of the nutritional status and PEW [19]. However, we failed to find it significantly correlated with muscle mass decline and the muscle decline rate in this study. The mean level of albumin in all the participants in our series was 40.1 ± 2.6 g/L. Interestingly, for patients suffering muscle decline, their albumin levels were not decreased significantly. Other nutritional markers including nPNA, hemoglobin, cholesterol, and MQSGA were also comparable between the NMM and MMD groups. This finding suggests that the classical nutritional markers may not be able to reflect muscle mass decline immediately. Muscle mass decline could occur before nutritional marker change and may be a strategy to assess the nutritional status.

Skeletal muscle is proved to be a target for 25(OH)D [20]. Studies showed that vitamin D supplement in adult rats helped cell regenerative process promotion in skeletal muscle after injury [21]. The positive association between 25(OH)D and muscle mass was also observed in human studies. Physical performance was found to be associated with the 25(OH)D concentration in an observational cohort study [6]. However, a meta-analysis involving a large number of randomized clinical trials showed that vitamin D supplement may slightly improve physical performances, but it may not be beneficial to muscle strength and muscle mass [22]. Although previous studies reported vitamin D supplementation increased muscle strength in MHD patients [23], few studies have reported the association between muscle mass decline and serum vitamin D level. The present study showed that serum 25(OH)D was an independent factor of muscle mass decline and muscle mass decline speed. Vitamin D may help preserve muscle mass and prevent muscle decline, but more randomized studies are needed to confirm the conclusion. The levels of other vitamins including vitamin C, vitamin E, vitamin B2, and vitamin B12 were lower in the high-rate group, implying that vitamins may be involved in muscle metabolism. However, 25(OH)D was found to be the only potential factor associated with muscle mass decline when adjusted for other indexes in univariate analysis. Further exploration is needed to evaluate the role of these vitamins in muscle mass and muscle functions.

B-type natriuretic peptides produced in the cardiac ventricles are promising markers of cardiac failure in general population [24]. NT-proBNP is the inactive fragment generated during BNP production which is widely used to adjust left ventricular hypertrophy and fluid overload [25]. A recent study showed that a high level of NT-proBNP was an independent predictor for decrease in lean body mass in MHD patients [26]. In the present study, patients in the high-rate group showed higher levels of NT-proBNP. NT-proBNP may not only assist in the diagnosis of heart failure and cardiac dysfunction but is a useful marker for identifying the risk of muscle mass decline in hemodialysis patients.

A strength of the present study is that we focused on the changes in muscle mass in MHD patients. We reported an association between age, 25(OH)D, dialysis duration, NT-proBNP, and muscle mass decline in MHD patients. It was worth noting that muscle mass decline could still occur when patients were considered to be in good nutritional status. Clinicians should be aware of patients who may be at risk for muscle mass decline.

However, there are some limitations. It was a retrospective study with limited number of participants. This sample may lack statistical power for analyzing the risk factors associated with muscle mass decline. Only patients who had two BIA measurements were included in this study. We could not exclude some degree of selection bias, and this cohort may not be a representative cohort for MHD patients. Comprehensive assessments of physical function such as short physical performance battery and usual gait speed are required. Further investigations should be performed to confirm whether vitamin D supplement helps muscle mass preservation.

## 5. Conclusion

Data obtained in our study support an association between 25(OH)D and NT-proBNP levels and muscle mass decline in prevalent hemodialysis patients. Future work is needed to delineate if assessment of 25(OH)D and NT-proBNP should be integrated into early diagnosis of PEW in hemodialysis patients.

## Figures and Tables

**Table 1:** Comparison of patient demographics, nutritional parameters, biochemical measures, vitamins, and anthropometric indices between the normal muscle mass group and the muscle mass declined group.

Parameters	Normal group (*n* = 25)	Declined group (*n* = 47)	*P*
*Patient demographics*			
Male (%)	10 (40.0)	23 (48.9)	.469
Age (y)	52.12 ± 12.49	59.20 ± 9.08	.007^∗^
Duration of dialysis (y)	8.01 ± 5.84	7.24 ± 4.88	.551
Kt/*v*	1.29 ± 0.21	1.38 ± 0.22	.094
*Primary disease*			
Glomerulonephritis (%)	6 (24.0)	23 (48.9)	.072
Hypertension (%)	3 (12.0)	10 (21.3)	.330
Diabetes (%)	6 (24.0)	5 (10.6)	.134
Others/unknown (%)	10 (40.0)	9 (19.1)	.103
*Complications*			
Cardiovascular disease	2 (8.0)	7 (14.9)	.400
Cerebrovascular events	3 (12.0)	6 (12.8)	.925
Severe inflammations	5 (20.0)	10 (21.3)	.899
Hospitalizations	14 (56.0)	30 (63.8)	.516
*Nutritional parameters*			
nPNA (g/kg/d)	1.11 ± 0.23	1.15 ± 0.24	.499
Serum albumin (g/L)	40.6 ± 3.3	39.9 ± 2.2	.265
Hemoglobin (g/L)	115.7 ± 15.5	110.1 ± 13.9	.237
HbA1c (%)	5.88 ± 0.81	5.78 ± 0.80	.586
Serum creatinine (*μ*mol/L)	969.82 ± 233.76	911.15 ± 202.27	.270
Cholesterol (mmol/L)	4.24 ± 0.70	4.30 ± 0.97	.792
MQSGA score	10.12 ± 1.22	11.17 ± 1.34	.144
*Biochemical measures*			
hs-CRP (mg/L)	1.34 (0.47, 5.31)	1.33 (0.56, 4.20)	.257
Log NT-proBNP	8.07 ± 1.01	7.98 ± 0.88	.674
CO_2_-CP (mmol/L)	20.67 ± 2.00	21.64 ± 2.88	.137
Ca (mmol/L)	2.40 ± 0.28	2.39 ± 0.30	.920
P (mmol/L)	1.83 ± 0.55	1.73 ± 0.49	.449
iPTH (pg/mL)	169.5 (97.0, 282.5)	253.5 (125.8, 357.0)	.723
*Vitamins*			
Vitamin A (*μ*mol/L)	1.16 ± 0.46	1.27 ± 0.55	.419
Vitamin B1 (nmol/L)	81.38 ± 17.97	80.69 ± 19.85	.885
Vitamin B2 (*μ*g/L)	318.05 ± 88.40	288.12 ± 59.94	.093
Vitamin B6 (*μ*mol/L)	31 ± 8.66	34.75 ± 10.61	.140
Vitamin B9 (nmol/L)	13.78 ± 3.78	13.22 ± 3.67	.547
Vitamin B12 (pg/mL)	383.99 ± 119.82	397.75 ± 148.41	.695
Vitamin C (*μ*mol/L)	54.04 ± 10.13	56.85 ± 15.07	.414
25(OH)D (nmol/L)	39.31 ± 9.97	33.17 ± 8.12	.006^∗^
Vitamin E (*μ*g/mL)	11.99 ± 1.39	12.28 ± 1.66	.474
*Anthropometric indices*			
Body fat rate (%)	21.49 ± 7.89	22.15 ± 8.28	.743
Waist/hip ratio	87.67 ± 6.58	87.17 ± 7.76	.821
ASM/H^2^ (kg/m^2^)	6.63 ± 1.07	6.51 ± 1.08	.663
MAMC (cm)			
Male	24.70 ± 1.98	24.60 ± 3.00	.923
Female	24.24 ± 2.60	21.57 ± 1.72	.012^∗^
Handgrip (kg)			
Male	32.21 ± 5.17	31.01 ± 9.84	.701
Female	20.11 ± 5.06	16.19 ± 5.47	.187
Daily steps	5852 ± 1723	4214 ± 2180	.157

Note: values indicate means ± SD, proportion, or median (IQR). nPNA: normalized protein equivalent of nitrogen appearance; MQSGA: modified quantitative subjective global assessment; hs-CRP: hypersensitive C-reactive protein; NT-proBNP: N-terminal prohormone of brain natriuretic peptide; CO_2_-CP: carbon dioxide combining power; iPTH: intact parathyroid hormone; ASM/H^2^: height-adjusted appendicular skeletal muscle mass; MAMC: midarm muscle circumference; ^∗^*P* < .05.

**Table 2:** Logistic regression analyses of the selected possible risk factors for muscle mass decline in MHD patients.

Variables	Univariate			Multivariable		
	OR	OR 95% CI	*P* value	OR	OR 95% CI	*P* value
Gender	0.885	0.335, 2.336	.805			
Age	1.068	1.016, 1.123	.010^∗^	1.077	1.015, 1.144	.014^∗^
Dialysis duration	0.972	0.886, 1.066	.546			
Albumin	0.891	0.727, 1.091	.264			
25(OH)D	0.921	0.865, 0.981	.011^∗^	0.931	0.869, 0.997	.040^∗^
hs-CRP	0.936	0.833, 1.052	.269			

Note: hs-CRP: hypersensitive C-reactive protein; ^∗^*P* < .05.

**Table 3:** Comparison of patient demographics, nutritional parameters, biochemical measures, vitamins, and muscle measures between the low- and high-rate groups.

Parameters	Low-rate group (*n* = 22)	High-rate group (*n* = 25)	*P*
*Patient demographics*			
Male (%)	8 (36.4)	15 (60.0)	.715
Age (y)	55.77 ± 7.68	62.40 ± 10.86	.062
Duration of dialysis (y)	5.30 ± 3.46	8.93 ± 5.36	.016^∗^
Kt/*v*	1.35 ± 0.21	1.32 ± 0.20	.234
Muscle decline rate (kg/m^2^/y)	0.11 ± 0.05	0.42 ± 0.20	.000^∗^
*Complications*			
Cardiovascular disease	2 (9.0)	5 (25.0)	.295
Cerebrovascular events	1 (4.5)	5 (25.0)	.113
Severe inflammations	6 (27.3)	4 (16.0)	.346
Hospitalizations	14 (63.6)	16 (64.0)	.979
*Nutritional parameters*			
nPNA (g/kg/d)	1.16 ± 0.21	1.14 ± 0.26	.822
Serum albumin (g/L)	40.5 ± 2.0	39.4 ± 2.2	.154
Hemoglobin (g/L)	109.9 ± 12.9	110.5 ± 14.8	.175
HbA1c (%)	5.60 ± 0.34	5.93 ± 1.04	.167
Serum creatinine (*μ*mol/L)	949.76 ± 203.39	877.16 ± 199.20	.248
Cholesterol (mmol/L)	4.43 ± 1.14	4.18 ± 0.79	.334
MQSGA score	11.13 ± 1.46	11.25 ± 1.16	.839
*Biochemical measures*			
hs-CRP (mg/L)	1.50 (0.67, 3.29)	0.85 (0.45, 4.99)	.987
Log NT-proBNP	7.59 ± 0.75	8.32 ± 0.85	.006^∗^
CO_2_-CP (mmol/L)	21.36 ± 2.64	21.89 ± 3.23	.486
Ca (mmol/L)	2.39 ± 0.28	2.39 ± 0.32	.983
P (mmol/L)	1.83 ± 0.45	1.63 ± 0.52	.198
iPTH (pg/mL)	259.0 (115.3, 443.3)	198.0 (121.3, 313.5)	.369
*Vitamins*			
Vitamin A (*μ*mol/L)	1.42 ± 0.55	1.14 ± 0.53	.067
Vitamin B1 (nmol/L)	78.75 ± 20.96	82.38 ± 19.07	.522
Vitamin B2 (*μ*g/L)	313.65 ± 58.14	265.64 ± 50.75	.018^∗^
Vitamin B6 (*μ*mol/L)	35.92 ± 9.55	33.72 ± 11.56	.458
Vitamin B9 (nmol/L)	440.45 ± 153.53	360.17 ± 135.92	.048
Vitamin B12 (pg/mL)	35.86 ± 7.61	30.78 ± 7.94	.048^∗^
Vitamin C (*μ*mol/L)	61.85 ± 16.75	52.44 ± 12.11	.017^∗^
25(OH)D (nmol/L)	35.86 ± 7.61	30.78 ± 7.94	.048^∗^
Vitamin E (*μ*g/mL)	12.77 ± 1.82	11.85 ± 1.43	.047^∗^
*Anthropometric indices*			
Body fat rate (%)	22.03 ± 7.07	22.27 ± 9.38	.919
Waist/hip ratio	88.65 ± 7.38	86.17 ± 8.76	.631
ASM/H^2^ (kg/m^2^)	6.29 ± 1.17	6.70 ± 0.98	.196
MAMC (cm)			
Male	25.80 ± 3.00	23.10 ± 2.59	.789
Female	21.12 ± 1.71	22.69 ± 1.30	.231
Handgrip (kg)			
Male	35.00 ± 11.40	26.03 ± 5.03	.078
Female	16.25 ± 4.50	16.15 ± 1.55	.963
Daily steps	4822 ± 2771	3672 ± 1338	.120

Note: values indicate means ± SDs, proportions, or median (IQR). nPNA: normalized protein equivalent of nitrogen appearance; MQSGA: modified quantitative subjective global assessment; hs-CRP: hypersensitive C-reactive protein; NT-proBNP: N-terminal prohormone of brain natriuretic peptide; CO_2_-CP: carbon dioxide combining power; iPTH: intact parathyroid hormone; ASM/H^2^: height-adjusted appendicular skeletal muscle mass; MAMC: midarm muscle circumference; ^∗^*P* < .05.

**Table 4:** Linear regression model predicting the ASM/H^2^ decline rate in MHD patients.

Variables	Single			Multivariable		
	Coefficient	95% CI	*P* value	Coefficient	95% CI	*P* value
Gender	0.126	0.003, 0.249	.047^∗^			
Age	0.007	0.001, 0.014	.053			
Dialysis duration	0.006	-0.002, 0.024	.008^∗^	0.011	0.001, 0.024	.030^∗^
Albumin	-0.029	-0.057, 0.021	.049^∗^			
Vitamin B2	-0.001	-0.002, 0.001	.027^∗^			
25(OH)D	-0.010	-0.018, 0.005	.018^∗^	-0.009	-0.016, -0.003	.007^∗^
Log NT-proBNP	0.237	0.101, 0.373	.040^∗^	0.106	0.011, 0.123	.020^∗^
Vitamin B12	0.001	-0.001, 0.001	.062			
Vitamin C	0.002	-0.008, 0.005	.096			
Vitamin E	0.019	-0.074, 0.021	.055			

Note: NT-proBNP: N-terminal prohormone of brain natriuretic peptide; ^∗^*P* < .05.

## Data Availability

All data included in this study are available upon request by contact with the corresponding authors.
